# Preparation of Concentrated PMMA Suspensions Stabilized by a Green Polysiloxane Surfactant

**DOI:** 10.3390/polym17182535

**Published:** 2025-09-19

**Authors:** Diana Borisova, Kirill Borisov, Aleksandra Kalinina, Aleksandra Bystrova, Inessa Gritskova, Aziz Muzafarov

**Affiliations:** 1 Enikolopov Institute of Synthetic Polymeric Materials, Russian Academy of Sciences, Moscow 117393, Russiakalinina@ispm.ru (A.K.);; 2 A.N. Nesmeyanov Institute of Organoelement Compounds, Russian Academy of Sciences, Moscow 119334, Russia; 3 Department of Chemistry and Technology of Macromolecular Compounds, MIREA—Russian Technological University, Moscow 119454, Russia

**Keywords:** suspension, polymethylsilsesquioxanes, hyperbranched polymers, polymerization, surfactant, PMMA

## Abstract

This study presents an approach to stabilizing suspension particles using novel eco-friendly hyperbranched organosilicon surfactants—poly(methyl ethoxysiloxane) with poly(ethylene glycol) groups (PMEOS-PEG). The surface-active properties of PMEOS-PEG polymers at the methyl methacrylate–water interface were thoroughly investigated. We demonstrate the successful preparation of concentrated, stable aqueous suspensions of poly(methyl methacrylate) with tunable particle sizes ranging from 370 nm to 840 nm.

## 1. Introduction

In recent decades, aqueous polymer dispersions (APDs) have gained widespread global adoption as environmental regulations have significantly limited emissions of volatile organic compounds in the production of coatings, adhesives, inks, and other applications [1,2,3]. According to statistical data [4,5], approximately 7.5–8 million metric tons of APDs are produced and sold worldwide annually, with this figure steadily increasing each year. Among these, acrylic dispersions—particularly monodisperse poly(methyl methacrylate) (PMMA) microspheres—have become increasingly prevalent due to their excellent transparency, biocompatibility, low cost, and other advantages [6,7]. The modifiable ester group in PMMA has enabled its use in diverse applications, including coatings, paper and textile adhesives, and functional finishing materials [7,8].

PMMA microspheres can be synthesized via various radical polymerization methods [9,10]. Industrially, however, heterophase polymerization in aqueous media is preferred as an environmentally benign approach for producing high-molecular-weight microspheres [11]. While water-based heterophase polymerization is inherently eco-friendly, its sustainability is compromised by the toxicity of conventional surfactants—such as fatty acid salts and aromatic/aliphatic sulfonic acid salts—and challenges in waste treatment. These surfactants accumulate in wastewater and nearby water bodies due to poor degradability [12].

Additional drawbacks of emulsion polymerization include coagulum formation during the reaction and polymer deposition on reactor surfaces. To mitigate these issues, fractional dosing of monomers or surfactants is employed, though this requires complex equipment and reduces process reproducibility [13].

Over the past twenty years, a shift has emerged toward utilizing organosilicon oligomers as alternatives to conventional organic surfactants. These silicone-based surfactants are biologically inert and environmentally benign.

Amphiphilic organosilicon surfactants consist of two structural blocks: hydrophobic polydimethylsiloxane (PDMS) fragments and attached polar groups. Due to the unique architecture of their polysiloxane backbone [14], they often outperform traditional surfactants in efficiency. For instance, they reduce surface tension from 30 mN/m (typical for hydrocarbon surfactants) to 20 mN/m [15].

Notably, silicone surfactants exhibit surface activity in both aqueous and non-aqueous media [16], as well as in supercritical CO_2_ [17].

Silicone surfactants form adsorption layers of high strength and viscosity, which influence the monomer dispersion process [13]. When monomer droplets are stabilized by silicone surfactants, they undergo less dispersion compared to droplets stabilized by water-soluble surfactants. Emulsion stabilization occurs at lower concentrations of silicone surfactants. The system’s stability at all stages of polymerization is ensured by the absence of stabilizer desorption during the transformation of the liquid/liquid interface into a liquid/polymer (swollen with monomer) interface during the formation of polymer-monomer particles (PMPs) [15].

Additionally, silicone oligomers containing reactive vinyl, vinylbenzyl, and methacrylic groups can act as both surfactants and comonomers during polymerization, forming stable suspensions at surfactant concentrations 5–6 times lower than those required for water-soluble ionic surfactants like sodium dodecyl sulfonate [18].

Studies [19,20] have thoroughly investigated the influence of linear, comb-like, and dimeric silicone surfactants on polymerization kinetics, particle size, and emulsion stability.

Hyperbranched polyethoxysiloxanes (PEOS) have been used as surfactants, enabling the formation of stable polystyrene and PMMA suspensions with a silica shell and narrow particle size distribution [21,22,23,24]. A key feature of this approach is the reactivity of PEOS, which acts both as a precursor for the silica shell on polymer particles and as a surfactant. Its amphiphilicity arises from the hydrolysis of hydrophobic ethoxy groups, generating hydroxyl groups. For instance, a study [21] demonstrated that using a PEOS/styrene mixture at a 1:400 ratio reduces the interfacial tension with water from 30 mN/m (pure styrene) to 11 mN/m. In contrast, for PMMA particles, the interfacial tension at the MMA/PEOS–water boundary only decreased from 12–13 mN/m to 8 mN/m [22]. Similarly, using PEOS as a surfactant, water/styrene/water double emulsions were obtained, leading to polystyrene–silica particles with complex structures [23].

Preliminary modification of PEOS with polyethylene glycol (PEOS-PEG) significantly reduces the interfacial tension of the PEOS-PEG/styrene mixture with water to extremely low values (0.05 mN/m) at ~17% PEOS-PEG content in styrene [24].

Previously, we reported the synthesis of hyperbranched polymethylsilsesquioxanes modified with varying amounts of polyethylene glycol (PMEOS-PEG) [25]. These contain hydrophobic methyl substituents on silicon, preserving their amphiphilicity even after the hydrolysis of ethoxy and PEG groups due to the presence of both hydrophilic hydroxyl and hydrophobic methyl groups. This contrasts with PEOS-PEG, which forms silica containing only hydroxyl groups.

The aim of this work was to study the colloid-chemical properties of PMEOS-PEG at the MMA/water interface and evaluate their potential as surfactants for producing concentrated (30–50%), storage-stable PMMA suspensions. Additionally, we examined the influence of PEG substituents on particle size and stability.

## 2. Materials and Methods

### 2.1. Materials

Methyltriethoxysilane (99%, Reachem, Moscow, Russia) was distilled under argon prior to use. Methyl methacrylate (MMA) (99.0%, Reachem, Moscow, Russia) was purified by double vacuum distillation. Acetic acid (99%, Component-Reaktiv, Moscow, Russia) and ethanol were dried via prolonged boiling followed by distillation over P_2_O_5_ under argon. The following reagents were used as received: sodium hydroxide (99%, Component-Reaktiv, Moscow, Russia), aqueous ammonia solution (25%, SIGMATEK, Khimki, Russia), poly(ethylene glycol) monomethyl ether (average molecular weight 350, ABCR, Karlsruhe, Germany), potassium persulfate (PP) (99.9%, Reachem, Moscow, Russia), and hexane (99%, Component-Reaktiv, Moscow, Russia). Deionized water was used throughout the experiments.

### 2.2. Methods

**Synthesis of hyperbranched polymethylethoxysiloxane (PMEOS)**. PMEOS was synthesized according to the previously reported procedure [26]. ^1^H NMR (CDCl_3_, δ, ppm): 3.74–3.82 (m, 2H, Si–OCH_2_CH_3_), 1.21 (t, 3H, Si–OCH_2_CH_3_),0.08–0.2 (m, 3H, Si–CH_3_)

**PEGylation of PMEOS**. PMEOS-PEG with three PEGylation degrees (4%, 9%, and 21%) was prepared as described in our earlier work [25] (see Table 1 for loadings and yields). ^1^H NMR (CDCl_3_, δ, ppm): 3.74–3.82 (m, 2H, Si–OCH_2_CH_3_), 3.66–3.62 (m, 4H, OCH_2_CH_2_), 3.37 (s, 3H, C-O(CH_3_)), 1.21 (t, 3H, Si–OCH_2_CH_3_),0.08–0.2 (m, 3H, Si–CH_3_).

^1^H NMR spectra of PMEOS-PEG (Figure S1) and GPC curves of PMEOS-PEG (Figure S2) are provided in the Supplementary Materials.

**Suspension Preparation.** MMA and deionized water were separately degassed by two freeze–pump–thaw cycles under reduced pressure (p = 10^−3^–10^−4^ mmHg) to remove dissolved air. Two phases were then prepared in a closed system: an oil phase, consisting of monomer (MMA) and PMEOS-PEG (1–5 wt.% relative to monomer), and an aqueous phase, consisting of water and dissolved initiator (K_2_S_2_O_8_). The oil phase was injected into the aqueous phase via syringe. The monomer-to-water volume ratio was varied from 1:1 to 1:4. The mixture was emulsified for 1 min to form a stable milky emulsion, then the flask was equipped with a reflux condenser and calcium chloride tube and heterophase polymerization was conducted at 85 ± 0.5 °C (oil bath) with stirring (300 rpm) for 2 h. Monomer conversion (P, %) was determined gravimetrically after adding the polymerization inhibitor hydroquinone. For more information about the definition of monomer conversion, see the Supplementary Materials. GPC curves of the dried polymer suspensions (Figure S3) are provided in the Supplementary Materials.

*GPC Analysis*: Gel-permeation chromatography (GPC) was performed on a chromatographic system consisting of a STAIER series II high-pressure pump (Aquilon, Nakhodka, Russia), a RIDK 102 refractometric detector (all Laboratory Apparatus Prague, Prague, Czech Republic), and a JETSTREAM 2 PLUS column thermostat (KNAUER, Berlin, Germany). The temperature was controlled at 40 °C (±0.1 °C). Tetrahydrofuran was used as the eluent, the flow rate was 1.0 mL/min. A 300 ± 7.8 mm column filled with Phenogel sorbent (Phenomenex, Torrance, CA, USA) with a particle size of 5 μm and a pore size of 103 Å was used (passport separation range—up to 75,000 D). Recording and processing of data was carried out using UniChrom 4.7 software (Minsk, Belarus).

*^1^H NMR Spectroscopy*: ^1^H NMR spectra were acquired using a Bruker WP250 SY spectrometer (Bruker, Karlsruhe, Germany) with CDCl_3_ as the solvent.

*Interfacial Tension (IFT*, *σ*, *mN/m)*: Measured using a Krüss spinning drop tensiometer at 25 °C (Krüss, Hamburg, Germany).

*Scanning Electron Microscopy (SEM)*: Conducted on a JEOL JCM-6000 PLUS microscope (JEOL, Tokyo, Japan) with an energy-dispersive spectrometer (5–15 kV accelerating voltage).

*Dynamic Light Scattering (DLS)*: Hydrodynamic diameter and ζ-potential of latex particles were determined using a Malvern Zetasizer Ultra at 25 °C (Malvern Panalytical GmbH, Worcestershire, UK).

Interfacial activity (g, mJ·m/mol) was determined from the slope of the IFT isotherm at the solvent’s surface tension. Critical aggregation concentration (CAC, wt.%) and maximum Gibbs adsorption (Γ_max_, mol/m^2^) were derived from plots of IFT vs. log[concentration] and Gibbs adsorption vs. concentration, respectively. Gibbs adsorption (Γ, mol/m^2^), molecular area (S_0_, Å^2^), and interfacial layer thickness (δ, nm) were calculated using Equations (S5)–(S7) (Supplementary Materials).

## 3. Results and Discussion

### 3.1. Colloid-Chemical Properties of PMEOS-PEGs at the MMA/Water Interface

The study began with an investigation of the colloid-chemical properties of the synthesized surfactants. All silicone-based surfactants were found to form direct oil-in-water (O/W) emulsions. Hyperbranched structure of our polymeric surfactants leads to denser molecular packing at the interface and, consequently, a more pronounced reduction in interface tension compared to their linear analogs [15]. The multiple functional groups located on the periphery of the macromolecule provide numerous interaction points with both phases, thereby enhancing adsorption at the interface.

**Figure 1:**
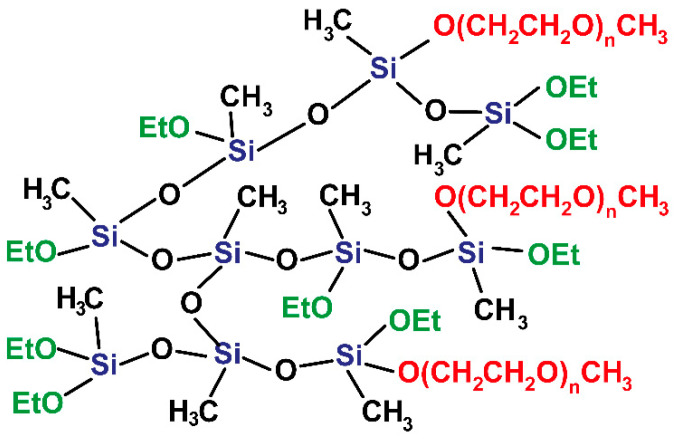
Chemical structure of PMEOS-PEG surfactants.

The general chemical structure of the synthesized PMEOS-PEGs is shown in Figure 1. The key characteristics—including molecular weight (M_n_), polydispersity (M_w_/M_n_), density, and hydrophilic–lipophilic balance (HLB), calculated via Griffin’s method [27] according to formula:

HLB = 20 × M_PEG_/M, (1)

—are summarized in Table 2.

**Figure 2:**
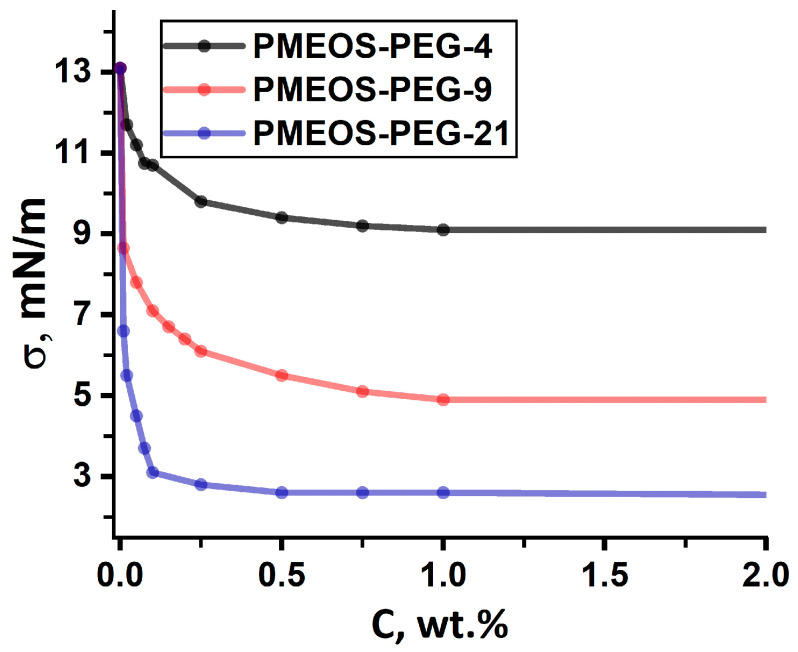
IFT isotherms at the MMA/water interface as a function of surfactant concentration in MMA.

Prior work [25] demonstrated the high interfacial activity of PMEOS-PEG at water/hexane and water/toluene interfaces. Here, we show that PMEOS-PEG also significantly reduces interfacial tension at the MMA/water interface (Figure 2).

PMEOS-PEG-4 reduced IFT from 13 mN/m to 9.1 mN/m at 1 wt.%. Increasing PEG content enhanced this effect: PMEOS-PEG-9 and PMEOS-PEG-21 lowered IFT to 4.9 mN/m and 2.4 mN/m, respectively, at just 1 wt.% (Figure 2).

The colloidal-chemical characteristics of the studied surfactants, namely, minimum interface tension value (σ, mN/m), interfacial activity (g, mJ·m/mol), maximum Gibbs adsorption (Г_max_, mol/m^2^), the area occupied by the surfactant molecule (S_0_, Å^2^), and the thickness of the interfacial layer formed by the surfactant molecules (δ, nm), are shown in Table 3.

Table 3 shows that an increase in the content of PEG substituents in PMEOS-PEG leads to enhanced interfacial activity and higher maximum adsorption (see also Figure S5), which correlates well with the increase in HLB values of PMEOS-PEG (Table 2). Due to the relatively high polarity of methyl methacrylate (MMA), effective reduction in interfacial tension at the MMA–water interface requires the use of surfactants with sufficiently high HLB values. At the same time, the area occupied by a surfactant molecule at the interface decreases. This behavior can be explained by two main factors. First, PMEOS-PEG-4 is strongly solvated by the relatively large MMA molecules, which create steric hindrance that prevents close packing of the PMEOS-PEG-4 molecules. As a result, the interfacial area occupied by each molecule is larger compared to PMEOS-PEG-9 and PMEOS-PEG-21, which possess significantly higher polarity, and therefore a larger proportion of their macromolecules are solvated by water. Second, with higher PEG content, the entropic factor associated with solvation in water increases. The PEG chains tend to extend into the aqueous phase to reduce the probability of their mutual proximity, further decreasing the area occupied by a single surfactant macromolecule. For the same reason, an increase in the PEG content results in a thicker adsorption layer.

### 3.2. Synthesis of PMMA Suspensions

**Figure 3:**
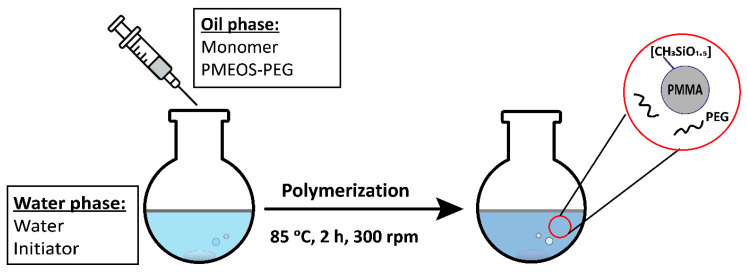
Schematic of PMMA suspension synthesis.

**Figure 4:**
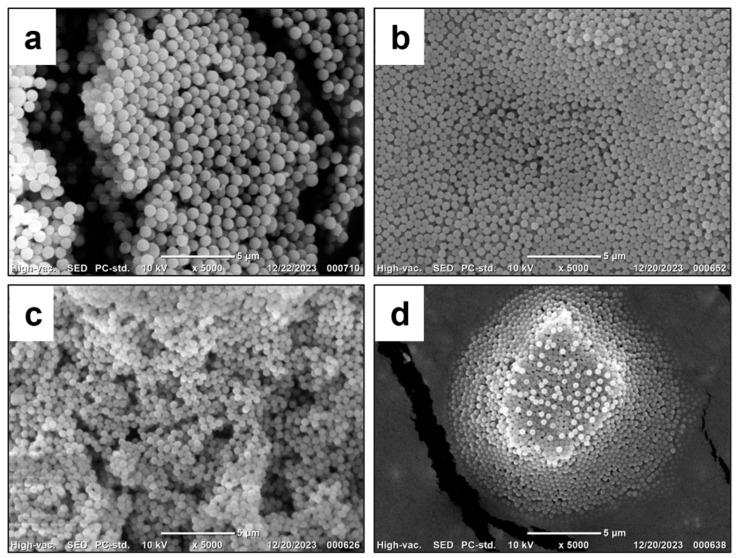
SEM images of the obtained PMMA particles stabilized with PMEOS-PEG-4: sample (**a**) No. 2, (**b**) No. 5, (**c**) No. 9, and (**d**) No. 16 from Table 4.

**Figure 5:**
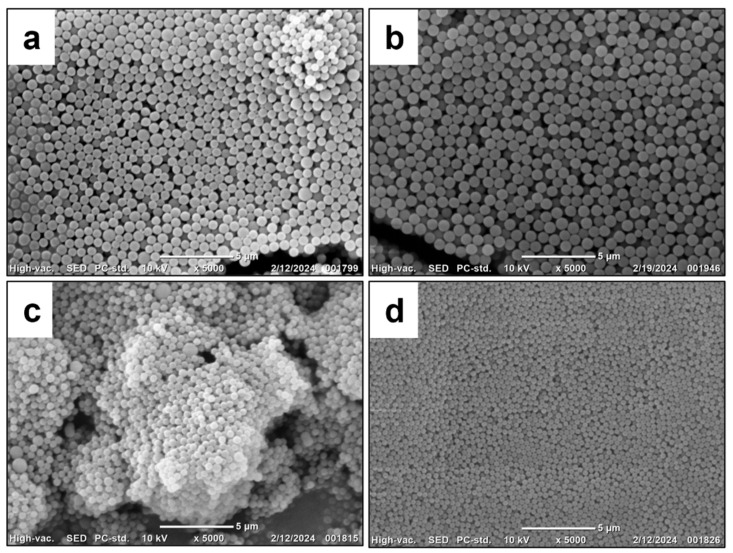
SEM images of the obtained PMMA particles stabilized with PMEOS-PEG-9: sample (**a**) No. 3, (**b**) No. 6, (**c**) No. 10, and (**d**) No. 17 from Table 4.

**Figure 6:**
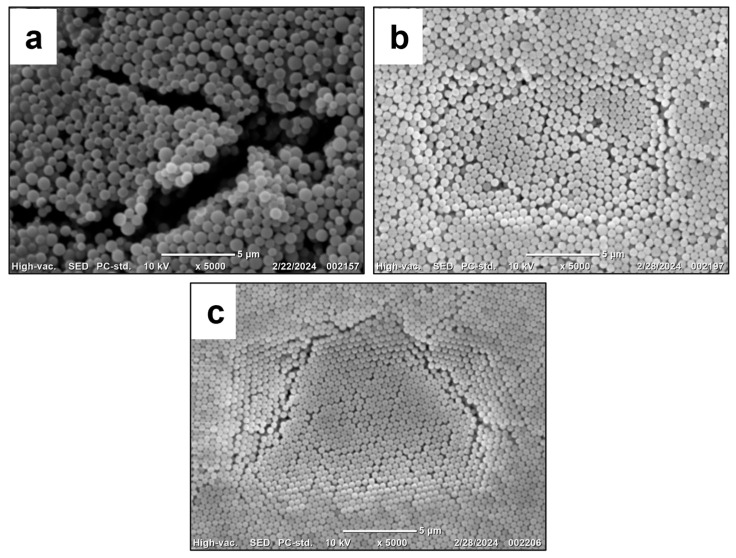
SEM images of the obtained PMMA particles stabilized with PMEOS-PEG-21: sample (**a**) No. 4, (**b**) No. 11, (**c**) No. 18 from Table 4.

Hyperbranched PMEOS-PEG surfactants containing 4–21 mol% PEG were tested as stabilizers in the heterophase polymerization of methyl methacrylate (MMA), initiated by potassium persulfate (PP, 1 wt.% relative to the monomer). The surfactant was first dissolved in MMA and then combined with the aqueous phase. Two surfactant loadings (C_sur_)—1 wt.% and 5 wt.% relative to the monomer—were examined. The monomer-to-water ratio was varied from 1:1 to 1:4, corresponding to polymer contents ranging from 20 to 50 wt.%. Polymerization was carried out at 85 ± 0.5 °C for 2 h under constant stirring at 300 rpm (Figure 3). During the reaction, the mixture’s color changed from pale blue to milky white, indicating the formation of particles with diameters exceeding 100 nm. The key characteristics of the resulting stabilized PMMA suspensions are summarized in Table 4 and illustrated in Figure 4, Figure 5 and Figure 6.

According to the data presented in Table 4, the use of 5 wt.% PMEOS-PEG as a surfactant—regardless of the PEG content—leads to the formation of concentrated (50% and 33%) polymer suspensions (samples No. 5–7 and No. 12–14 in Table 4, respectively). These suspensions contain 50% and 33% polymer and exhibit narrow particle size distributions, with particle diameters ranging from 460 to 840 nm for the 50% suspensions and from 590 to 740 nm for the 33% suspensions. The narrow particle size distribution is confirmed by the PDI_DLS_ values and by the SEM images of the obtained particles (Figure 4, Figure 5 and Figure 6).

Reducing the surfactant concentration to 1 wt.% in the case of 50% systems (samples No. 2–4 in Table 4) results in coagulum formation, preventing reliable measurement of particle size and monomer conversion (samples No. 1–4). For V_m_/V_H2O_ = 1:2 systems (samples No. 8–11, Table 4), a stable polymer suspension was obtained at 1 wt.% surfactant only when using PMEOS-PEG-21 (sample No. 11, Table 4), yielding a suspension with 33% polymer content and a particle size of 580 nm (Figure 6a). In the absence of any surfactant, concentrated (50% and 33%) polymer suspensions could not be obtained. This effect, observed with PMEOS-PEG-21, is attributed to its lower interfacial tension and higher interfacial activity compared to PMEOS-PEG-4 and PMEOS-PEG-9, which enables the formation of concentrated (33%) suspensions even at lower surfactant concentrations.

The particle diameter resulting from heterophase polymerization of MMA in the presence of the studied surfactants increases with the PEG content in PMEOS-PEG, a trend particularly pronounced in concentrated systems (Table 4). These findings deviate from the conventional trend in which increased PEG content (i.e., reduced interfacial tension) typically leads to smaller particle sizes. We attribute this behavior to two main factors. First, the hyperbranched, globular structure of the PMEOS-PEG surfactants likely requires a greater number of surfactant molecules to effectively stabilize smaller droplets [26,28]. Second, potassium persulfate initiates polymerization under acidic conditions, which leads to the hydrolysis of PEG fragments and alkoxy groups, followed by condensation with the resulting hydroxyl groups. This promotes the formation of polymethylsilsesquioxane structures, whose concentration decreases with increasing PEG content in the surfactant. This may explain why use of PMEOS-PEG-21, with its higher PEG content and smaller molecular area in the interfacial layer, results in larger particles than PMEOS-PEG-4 and PMEOS-PEG-9. Furthermore, in more dilute systems with a monomer-to-water phase ratio of 1:4, the influence of PEG content in PMEOS-PEG on particle size becomes negligible.

The negative zeta potential of the polymer particle surface arises from the use of potassium persulfate as the initiator, which decomposes to form sulfate ions. Since the number of functional groups on the surfactant molecules is much lower than that of the generated sulfate groups, the zeta potential is primarily determined by the amount of potassium persulfate used [29]. Because the colloidal stability of suspensions depends directly on electrostatic repulsion governed by the electric double layer, zeta potential values greater than ±30 mV indicate high colloidal stability [30]. As shown in Table 4, the zeta potential values range from −48.5 to −57.3 mV, indicating strong colloidal stability of the obtained suspensions.

**Figure 7:**
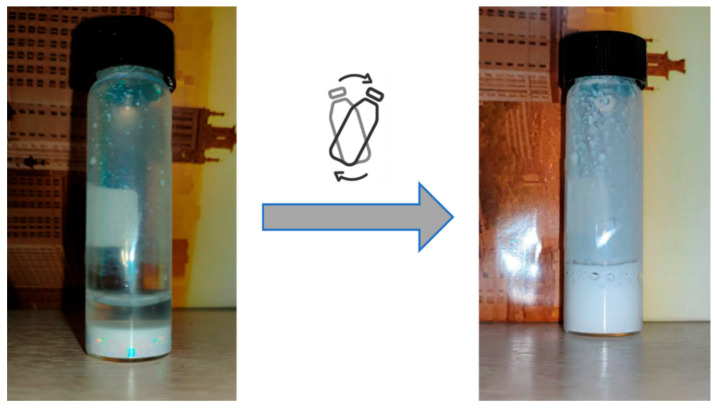
Illustration of particle redistribution after shaking of fully sedimented particles (sample No 13, Table 4).

It should be noted that the kinetic (sedimentation) stability of the suspensions depends directly on the size and shape of the particles [31]. Given their relatively large diameters, the particles are not effectively stabilized against gravitational settling. As a result, the suspensions remain stable for 3 to 5 days, depending on particle size: those with diameters >500 nm settle within 3 days, while particles in the 300–500 nm range settle within 5 days. However, due to their good colloidal stability, the particles can be easily redispersed throughout the suspension volume by simple stirring. After 3 months of storage, no significant changes in either particle diameter or zeta potential were observed (Figure 7 and Table 5).

**Figure 8:**
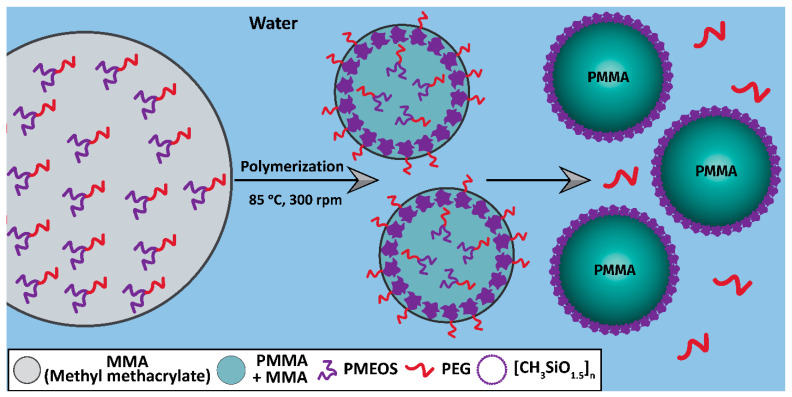
Schematic illustration of the stabilization mechanism for PMMA particles.

Figure 8 illustrates the mechanism of polymer particle formation in the presence of PEGylated hyperbranched organosilicon surfactants. The PMEOS-PEG-4, -9, and -21 surfactants are highly soluble in methyl methacrylate. With increasing PEGylation degree, the surfactant molecules migrate more intensively to the MMA/water interface, as evidenced by the lower interfacial tension (σ) values observed for PMEOS-PEG-21 compared to PMEOS-PEG-4. PMEOS-PEG-4 and PMEOS-PEG-9 are more hydrophobic; thus, their molecules are located not only at the interface but also within the bulk monomer phase.

Upon initiation of polymerization, phase separation occurs within the emulsion droplets, and surfactant molecules continuously diffuse to the interface, leading to local saturation. Hydrolysis of potassium persulfate acidifies the medium, which significantly reduces the hydrolysis time of surfactant molecules at the MMA/water interface. Acidic hydrolysis liberates the PEG chains, which migrate into the aqueous phase, while the PMEOS core hydrolyzes to [MeSiO_1.5_]_n_, which subsequently forms a thin shell at the surface of the PMMA particles.

## 4. Conclusions

In summary, we successfully synthesized concentrated PMMA suspensions (~50%) stabilized by 5 wt.% of hyperbranched organosilicon surfactants PMEOS-PEG-4, PMEOS-PEG-9, and PMEOS-PEG-21, with particle diameters ranging from 370 to 840 nm. The resulting particles exhibited narrow size distributions and remained stable during storage for all three surfactants.

The use of hyperbranched PEG-functionalized organosilicon surfactants revealed the following trend: in concentrated systems with monomer-to-water ratios of 1:1 and 1:2, an increase in PEG content (and thus in HLB value) led to an increase in particle diameter. In contrast, for more dilute systems (1:4), no clear dependence of particle size on PEG content was observed.

Importantly, it was demonstrated that a concentrated (33%) suspension with a particle size of 580 nm can be obtained using only 1 wt.% of PMEOS-PEG-21. In comparison, under the same conditions, systems without surfactant or with PMEOS-PEG-4 and PMEOS-PEG-9 produced significant amounts of coagulum.

This work highlights the utility of hyperbranched silicone surfactants for tailored polymer colloids.

## Figures and Tables

**Table 1:** Reaction conditions for the synthesis of PMEOS-PEG-4, -9, and -21.

Product	Ethoxy Group Substitution (mol%)	Mass of PMEOS (g)	Mass of PEG (g)	Yield (%)—(g)
PMEOS-PEG-4	4	25	2.8	99—43.7
PMEOS-PEG-9	9	25	7.0	99—47.5
PMEOS-PEG-21	21	25	14.5	99—55.2

**Table 2:** Characteristics of PMEOS-PEGs.

Sample	M_n(GPC)_	M_w_/M_n_	Density, g/cm^3^	HLB
PMEOS-PEG-4	1400	2.2	1.10	2.4
PMEOS-PEG-9	1700	2.2	1.15	4.6
PMEOS-PEG-21	2000	1.7	1.11	8.4

**Table 3:** Interfacial properties of PMEOS-PEGs at the MMA/water interface.

Surfactant	σ, mN/m	CAC, wt.%	Г_max_ × 10^7^, mol/m^2^	g, (mJ·m)/mol	S_0_, Å^2^	δ, nm
PMEOS-PEG-4	9.1	1	3.57	8.92	465	0.5
PMEOS-PEG-9	4.9	1	4.46	39.28	372	0.7
PMEOS-PEG-21	2.4	0.2	6.47	120.97	257	1.2

**Table 4:** Key characteristics of obtained PMMA suspensions.

Sample No.	Surfactant	V_m_/V_H2O_	C_sur_, wt.%	D_dls_ ± SD, nm	D_sem_ ± SD, nm	PDI_DLS_	ζ, mV	P, %
1	No surfactant	1:1	-	Coagulation				
2	PMEOS-PEG-4	1:1	1					
3	PMEOS-PEG-9	1:1						
4	PMEOS-PEG-21	1:1						
5	PMEOS-PEG-4	1:1	5	460 ± 10	490 ± 30	0.011	−57.7	91
6	PMEOS-PEG-9	1:1		530 ± 40	690 ± 50	0.008	−54.5	92
7	PMEOS-PEG-21	1:1		840 ± 60	790 ± 80	0.027	−51.7	94
8	No surfactant	1:2	-	Coagulation				
9	PMEOS-PEG-4	1:2	1					
10	PMEOS-PEG-9	1:2						
11	PMEOS-PEG-21	1:2		580 ± 110	560 ± 40	0.038	−52.6	98
12	PMEOS-PEG-4	1:2	5	590 ± 160	720 ± 60	0.073	−55.4	95
13	PMEOS-PEG-9	1:2		590 ± 60	500 ± 30	0.011	−55.9	92
14	PMEOS-PEG-21	1:2		740 ± 120	510 ± 30	0.024	−57.3	90
15	No surfactant	1:4	-	430 ± 110	350 ± 30	0.063	−50.7	88
16	PMEOS-PEG-4	1:4	1	370 ± 20	310 ± 20	0.003	−51.5	98
17	PMEOS-PEG-9	1:4		510 ± 70	330 ± 50	0.021	−54.8	99
18	PMEOS-PEG-21	1:4		520 ± 130	390 ± 30	0.056	−53.2	94

D_sem_ ± SD particle size was determined by averaging the diameters of 500 particles from electron micrographs. V_m_/V_H2O_ refers to the volume ratio of the monomer to water phases. P, % represents monomer conversion. PDI_DLS_ is the polydispersity index calculated from dynamic light scattering (DLS) data using the formula PDI_DLS_ = (SD/D_i_)^2^, where SD is the standard deviation and D_i_ is the average diameter by intensity.

**Table 5:** PMMA suspensions—comparison of freshly prepared and 3-month stored samples (sample No 13, Table 4).

Sample	Di, nm	ζ, mV
Freshly prepared	590 ± 60	−55.9
After 3 months of storage	580 ± 30	−53.7

## Data Availability

Data is contained within the article or Supplementary Materials.

## Supplementary Materials

The following supporting information can be downloaded at https://www.mdpi.com/article/10.3390/polym17182535/s1, Figure S1: ^1^H NMR spectra of surfactants PMEOS-PEG-4, PMEOS-PEG-9, and PMEOS-PEG-21; Figure S2: GPC curves of surfactants PMEOS-PEG-4, PMEOS-PEG-9, and PMEOS-PEG-21; Figure S3: GPC curves of PMMA suspensions; Gravimetric method for determining monomer conversion; Determination of interfacial parameters: Gibbs adsorption (Гmax, mol/m^2^), molecular area in the interfacial layer (S_0_, Å^2^), interfacial layer thickness (δ, nm); Figure S4: Structure of the adsorption layer at the liquid–liquid interface; Figure S5: Plots of Gibbs adsorption versus monomer concentration.
