# Supplementary material for: Preparation of Concentrated PMMA Suspensions Stabilized by a Green Polysiloxane Surfactant
Source: Polymers (Basel). 2025 Sep 19;17(18):2535. doi: 10.3390/polym17182535 (PMC12473809; doi:10.3390/polym17182535)
Supplement: Supplementary file 1 [file polymers-17-02535-s001.zip › polymers-3847642-supplementary.pdf]

Supplementary Materials

Preparation of Concentrated PMMA Suspensions Stabilized by a Green Polysiloxane Surfactant

Diana Borisova [1], Kirill Borisov [1], Alexandra Kalinina [1], Aleksandra Bystrova [1,2], Inessa Gritskova [3] and Aziz Muzafarov [1,2,*]

[1] Enikolopov Institute of Synthetic Polymeric Materials, Russian Academy of Sciences, Moscow 117393, Russia; kalinina@ispm.ru (A.K.)
[2] A.N. Nesmeyanov Institute of Organoelement Compounds, Russian Academy of Sciences, Moscow 119334, Russia
[3] Department of Chemistry and Technology of Macromolecular Compounds, MIREA—Russian Technological University, Moscow 119454, Russia
* Correspondence: aziz@ispm.ru

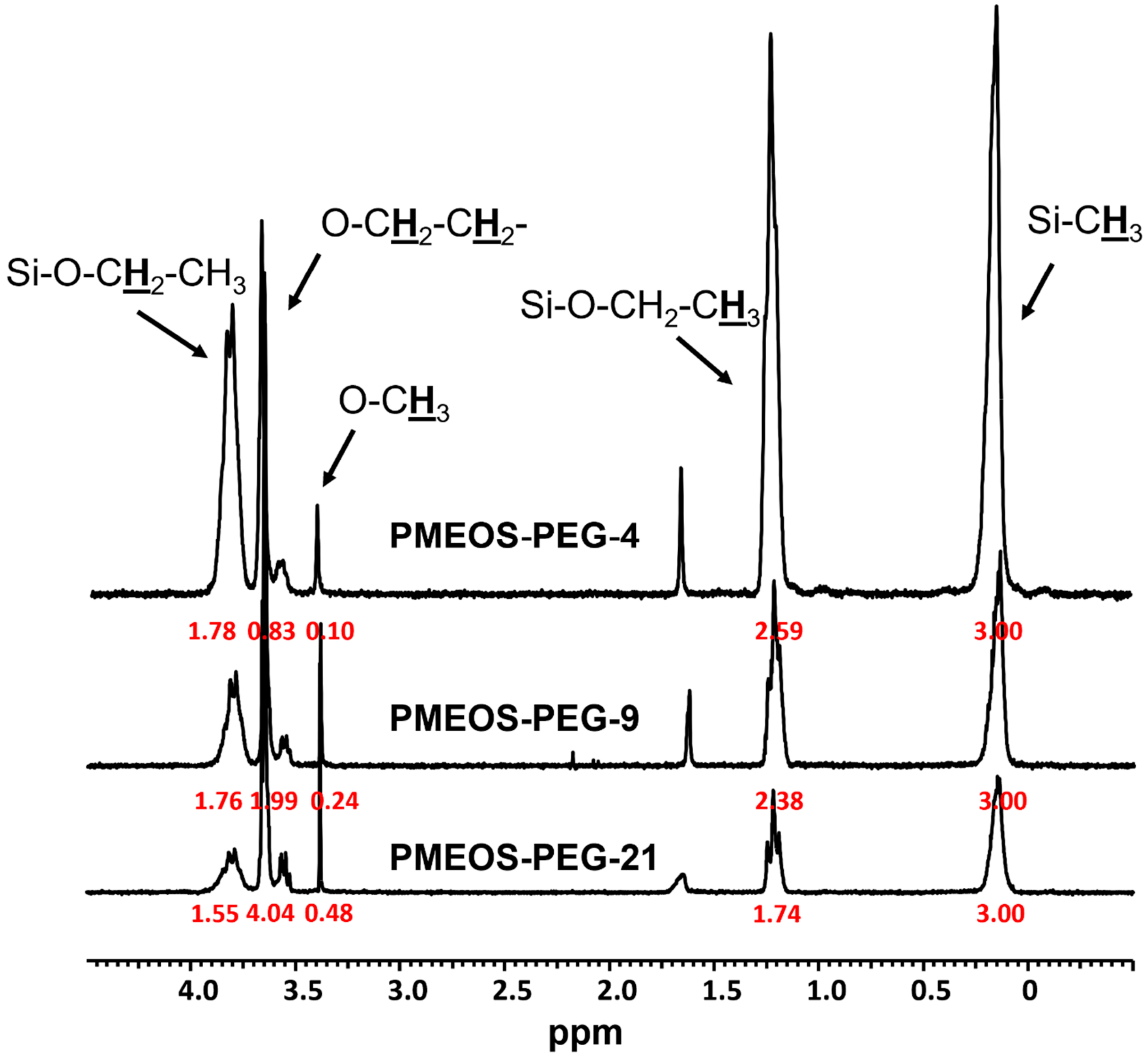

Figure S1. H NMR spectra of surfactants PMEOS-PEG-4, PMEOS-PEG-9, and PMEOS-PEG-21.

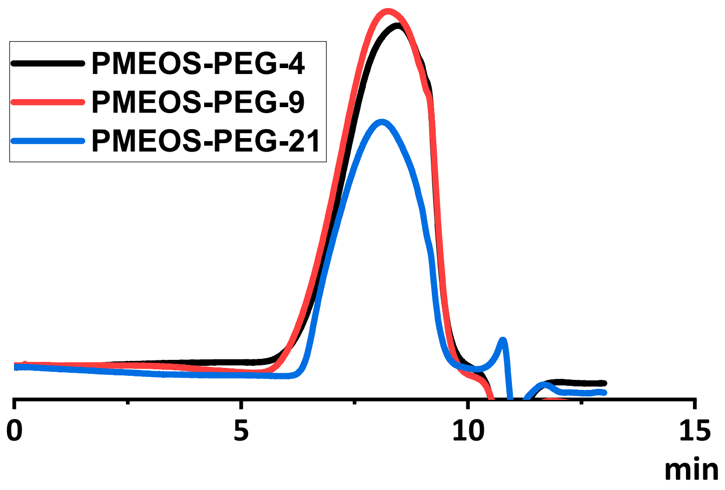

Figure S2—GPC curves of surfactants PMEOS-PEG-4, PMEOS-PEG-9, and PMEOS-PEG-21

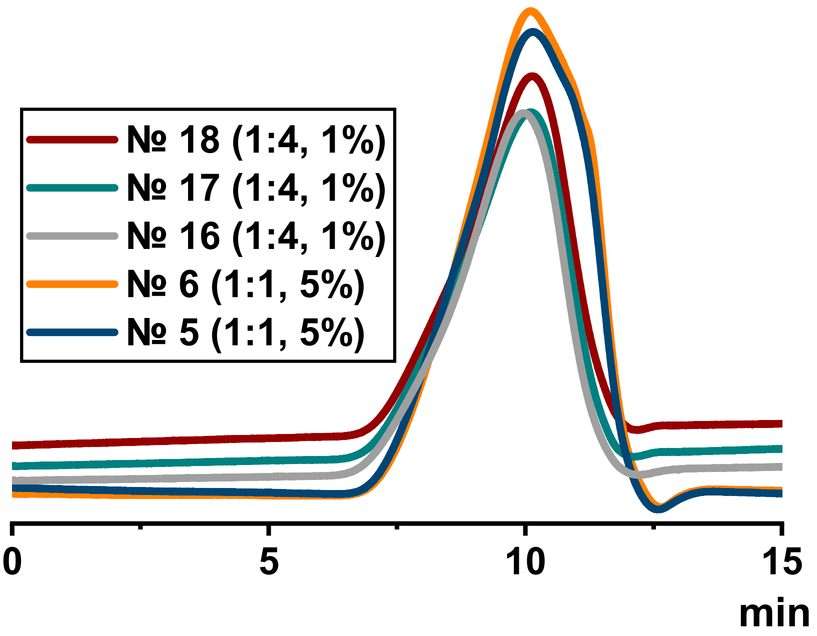

Figure S3—GPC curves of PMMA suspensions

Gravimetric method for determining monomer conversion

Into a pre-weighed Petri dish, 500 μL of the polymer suspension is placed and weighed. Then, 200 μL of a 4.3 wt.% aqueous solution of the polymerization inhibitor hydroquinone is added to it. The mixture is stirred, weighed (M), and placed in a drying oven for 1 hour at a temperature of 100–110 °C, and weighed again (M_{dry}). The mass

fraction of the polymer (solid content) relative to the amount of polymer suspension taken is calculated using the following Equation (S1):

$$X = \frac{\mathbf{M_{dry} - M_{inh}}}{\mathbf{M - M_{inh\,sol}}} \quad \mathbf{(S1)}$$

where M_{inh} and $M_{inh\ sol}$ are masses of the inhibitor and inhibitor solution, respectively.

The obtained value was correlated with the calculated solid content at 0% conversion ($X_{0\%}$), which corresponds to the absence of polymer, and with the calculated solid content at 100% conversion ($X_{100\%}$), where the mass of the obtained polymer equals the mass of the monomer used. The values of $X_{0\%}$ and $X_{100\%}$ were calculated using Equations (S2) and (S3), respectively.

$$X_{\mathbf{0\%}} = \frac{\mathbf{M_{in} + M_{surf}}}{\mathbf{M_{total}}} \quad \mathbf{(S2)}$$

$$X_{\mathbf{100\%}} = \frac{\mathbf{M_{in} + M_{surf} + M_{mon}}}{\mathbf{M_{total}}} \quad \mathbf{(S3)}$$

where M_{in}, M_{surf}, M_{mon}, and M_{total} are the masses of the initiator, surfactant, monomer, and total mass of the load (including water), respectively.

The monomer conversion, P, was subsequently calculated using Equation (S4):

$$\mathbf{P} = \frac{\mathbf{X - X_{0\%}}}{\boldsymbol{X}_{\mathbf{100\%}} - \mathbf{X_{0\%}}} \times \mathbf{100\%} \quad \mathbf{(S4)}$$

Determination of interfacial parameters:

Gibbs adsorption (Γ_{max}, mol/m^2):

$$\Gamma = \frac{C_1}{R \cdot T} \cdot \frac{d\sigma}{dC} \quad \mathbf{(S5)}$$

where R—universal gas constant, R≈8,314 J/(mol·K); T—temperature, K; and C_1—concentration, where dσ/dC is evaluated.

Molecular area in the interfacial layer (S_0, Å^2):

$$\boldsymbol{S} = \frac{1}{\Gamma_{max} \cdot N_a} \quad \mathbf{(S6)}$$

where N_a —Avogadro number, $N_a = 6{,}022 \cdot 10^{23}$ mol^{-1}.

Interfacial layer thickness (δ, nm):

$$\boldsymbol{\delta} = \frac{\Gamma_{max}}{\rho \cdot \mathrm{M}} \qquad \textbf{(S7)}$$

where M—molecular weight of surfactant, g/mol; and ϱ—surfactant density, g/m^3.

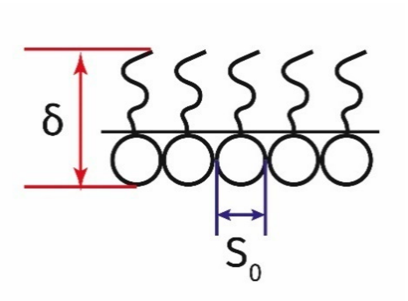

Figure S4—Structure of the adsorption layer at the liquid–liquid interface

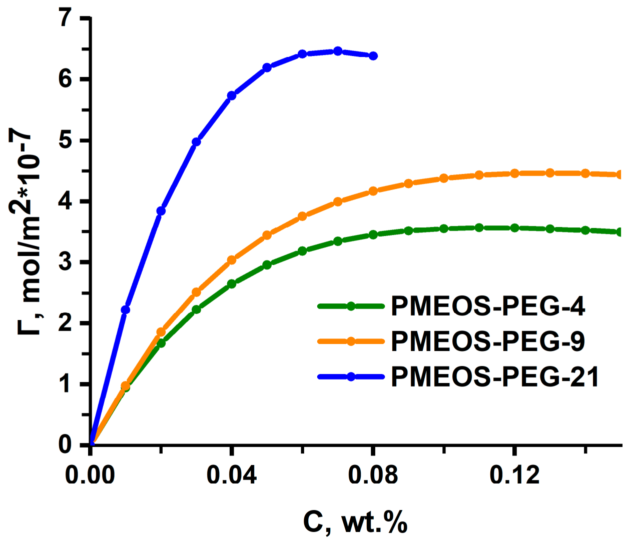

Figure S5—Plots of Gibbs adsorption versus monomer concentration
